# Total physical activity might not be a good measure in the relationship with HDL cholesterol and triglycerides in a multi-ethnic population: a cross-sectional study

**DOI:** 10.1186/1476-511X-10-223

**Published:** 2011-11-30

**Authors:** Jeroen SL de Munter, Irene G van Valkengoed, Karien Stronks, Charles Agyemang

**Affiliations:** 1 Academic Medical Center, Department of Public Health, University of Amsterdam, Amsterdam, The Netherlands

**Keywords:** epidemiology, physical activity intensity, physical activity duration, lipoprotein, African origin, South Asian origin, ethnic minority, high density lipoprotein, triglycerides

## Abstract

**Background:**

Evidence suggests that physical activity (PA) has a beneficial effect on high-density lipoprotein cholesterol (HDL) and triglycerides. However, observational studies show contrasting results for this association between different ethnic groups. It is unclear whether this is due to differences in the PA composition. The aim of this study was to assess the relationship of the total PA, along with its intensity and duration, with HDL and triglycerides in a multi-ethnic population.

**Methods:**

The study population was sampled from the SUNSET study and included: 502 European- Dutch, 338 Hindustani-Surinamese, and 596 African-Surinamese participants living in Amsterdam, the Netherlands. We assessed PA with the SQUASH questionnaire. We calculated age-sex-adjusted betas, geometric mean ratios (GMRs), and prevalence ratios (PRs) to assess the relationship of PA with HDL and triglycerides.

**Results:**

In the adjusted models, the highest total PA tertile compared to the lowest tertile was beneficially associated with HDL (beta: 0.08, 95% CI: 0.00, 0.16 and PR low HDL 0.59, 95% CI: 0.39, 0.88) and triglycerides (GMR: 0.93, 95% CI: 0.83, 1.03 and PR: 0.56, 95% CI: 0.29, 1.08) for the African-Surinamese. No statistically significant associations appeared for total PA among the European-Dutch and Hindustani-Surinamese. The adjusted models with the intensity score and HDL showed beneficial associations for the European-Dutch (beta: 0.06, 95% CI: 0.03, 0.10) and African-Surinamese (beta: 0.06, 0.02, 0.10), for log triglycerides for the European-Dutch (beta: -0.08, 95% CI: -0.12, 0.03), Hindustani-Surinamese (beta: -0.06, 95% CI: -0.16, 0.03), and African-Surinamese (beta: -0.04, 95% CI: -0.10, 0.01). Excepting HDL in African-Surinamese, the duration score was unrelated to HDL and triglycerides in any group.

**Conclusions:**

Activity intensity related beneficially to blood lipids in almost every ethnic group. The activity duration was unrelated to blood lipids, while the total PA 'summary score' was associated only with blood lipids for African-Surinamese. The difference in total PA composition is the most probable explanation for ethnic differences in the total PA association with blood lipids. Multi-ethnic observational studies should include not only a measure of the total PA, but other measures of PA as well, particularly the intensity of activity.

## Background

Physical activity (PA) is beneficially associated with blood lipids in European populations, especially HDL cholesterol and triglycerides [1-4]. Furthermore, this beneficial association appears as a clear dose-response association with blood lipids in controlled trials of European populations [5]. There is inconsistent evidence that this is the case in the few observational studies that include ethnic minority groups. For example, two recent multi-ethnic studies that looked into PA and blood lipids found associations that were different from previous evidence [6,7]. Black and white American participants from the ARIC study showed increases in PA that were associated with increases in HDL cholesterol for all participants, but decreases in triglycerides only for white participants. There was no correlation of HDL cholesterol levels with a PA index for either South Asian or white European adults in Newcastle, UK.

Explanations for these different patterns are unclear, but may be related to differences in the composition of PA between ethnic groups. Most habitual physical activities are of relative light intensity and long duration, such as light household activity, walking to work, walking in leisure time, or light occupational activity, which can differ between population groups [8]. These kinds of activities might weaken the effect of more intense activities that are known to be more beneficial in relation to HDL cholesterol and triglycerides especially when we try to incorporate all of them (including both vigorous and light activity) in one variable which we call 'total PA'. When patterns of PA differ between ethnic groups, such a measure of total PA might give differences in association with HDL cholesterol and triglycerides between ethnic groups.

The aim of this study was therefore to assess the relationship of total PA and the intensity and duration of PA with HDL cholesterol and triglycerides in a multi-ethnic study population living in Amsterdam, The Netherlands. More specifically, we tested whether the associations of intensity and duration of PA with HDL cholesterol and triglycerides in these ethnic groups lead to a more consistent pattern than the association of *total *PA with blood lipids.

## Methods

### Study population

We used data from the SUNSET study, which was set up to gain insight into the cardiovascular risk profiles of people aged 35-60 years in three ethnic groups living in Amsterdam, the Netherlands: Hindustani-Surinamese (South Asian origin), African-Surinamese (African origin) and European-Dutch origin. The recruitment and design of the study are described elsewhere [9,10]. In brief, potential participants were randomly sampled (*n *= 2975) from the population register of Amsterdam, The Netherlands. Potential participants - matched by sex and presumed ethnicity - were approached at home for a structured face-to-face interview with a trained interviewer between 2001 and 2003. The interview contained questions about lifestyle, migration history, demographic variables, and general health status. After the interview, the participants were invited for a physical examination at a local health centre. The response to the interview was 60% in the ethnic minority groups and 61% in the Dutch group. Only participants with complete information about blood lipids and ethnicity were included in this study, which resulted in a total of 1446 eligible participants, of whom there were 502 European-Dutch, 338 Hindustani-Surinamese, and 596 African-Surinamese. The Institutional Review Board of the Academic Medical Center of the University of Amsterdam approved the study. All participants provided written informed consent.

### Measurements

#### Main outcomes

We measured HDL cholesterol (homogenous enzymatic colorimetric test; Roche Diagnostics Basel, Switzerland) and triglycerides (GPO-PAP enzymatic test; Roche Diagnostics, Basel, Switzerland) in serum at the time of the physical examination. We defined low HDL cholesterol as less than 1.04 mmol/L (< 40.1 mg/dL) for men and less than 1.29 mmol/L (< 49.8 mg/dL) for women. We defined high triglyceride as 1.7 mmol/L (≥150 mg/dL) or more, or using cholesterol- or triglyceride-reducing medication [11].

#### Main predictor

We assessed the total habitual PA with a slightly modified SQUASH questionnaire [12]. These modifications have been described previously [13]. In short, the modifications included two extra leisure-time activities together with an open-ended question about any additional leisure-time PA. The SQUASH identifies types of activity grouped in *domains *of activities (transportation, work, household, and leisure time). For each activity, the respondent recalls the *component *frequency (days/week), duration (minutes/day), and intensity (low, moderate, or vigorous). We coded the type of activity in metabolic equivalents (METs) based on the Ainsworth compendium of PA [14]. The standard syntax calculates an overall PA score (total PA) and is based on frequency, duration, and intensity (including MET, age of respondent, and self-reported intensity) of the reported PA [12]. Additionally, we calculated MET-hours based on the MET and reported duration of activity. For this analysis, we also calculated the individual component sum scores for intensity and duration. We extracted these component scores exactly as they were used in calculating the total PA. Basically, for every reported activity, the intensity is calculated and summed to give the intensity score, and for every reported activity, the duration is summed to give the duration score.

#### Other covariates

The level of education was defined as primary school or less, secondary school, college or further education, and polytechnic or academic education. The smoking status was defined as never, ex, or current. Social class was defined on the Erikson et al. scale [15], and for the purpose of this study, condensed to six categories (I, II, IIIa, IIIb, IVa-c, and V-VI-VII combined into the sixth category). Alcohol intake was classed as never, ex, or current.

#### Analyses

We plotted the age-standardised levels (mean and median) and prevalence ratios (PRs) of HDL cholesterol and triglycerides in tertiles of the total PA score. We applied direct age standardisation, using the age distribution of the total study population. The triglyceride level was skewed, so we modelled log-transformed triglycerides in the adjusted models and presented back-transformed estimates (geometric mean ratios, GMRs). For the linear models of PA with HDL cholesterol and triglycerides, we excluded participants who reported lipid lowering medication use or any previous doctors' diagnoses of high cholesterol or raised levels of triglycerides.

We calculated age- and sex-adjusted models and further adjusted for education, smoking, alcohol intake, and social class to model the independent association of the total PA score on HDL cholesterol and triglycerides. To further verify the findings from the association of total PA with HDL cholesterol and triglycerides, we assessed the relationship of MET-hours and blood lipids across ethnic groups. We considered body weight an intermediate in the pathway from PA to HDL cholesterol and triglycerides. Since we were interested in the overall association between PA and blood lipids, but not the relative contribution of body mass index (BMI) on blood lipids, we excluded BMI in the models. Attempts to decompose the indirect association of BMI as an intermediate requires the absence of other confounding variables and homogeneity of these associations across the strata of the intermediate [16], which was beyond the scope of this paper.

We calculated age- and sex-adjusted models for the intensity score and duration score in association with HDL cholesterol and triglycerides. We presented this association as the difference in HDL cholesterol and triglycerides for one standard deviation increase in the intensity score and duration score. We took sex and ethnicity interactions into account. We used R (R: A Language and Environment for Statistical Computing, version 2.12.2) for all analyses. We considered values of *p *less than 0.05 to be statistically significant.

## Results

Table 1 presents the characteristics of the study population by ethnic group. The Hindustani-Surinamese and African-Surinamese people were younger, had a lower prevalence of smoking, and a lower level of education than the European-Dutch participants.

**Table 1:** Characteristics of 34 to 60-year-old Hindustani-Surinamese, African-Surinamese, and European-Dutch participants

	European-Dutch *n *= 502	Hindustani-Surinamese *n *= 338	African-Surinamese *n *= 596
Mean age (SD)	47.8 (6.8)	44.7 (6.7)	43.7 (5.9)
Women, *n *(%)	254 (50.6)	188 (55.6)	403 (67.6)
Never smokers, *n *(%)	128 (25.7)	167 (50.6)	250 (43.4)
Ex-smokers, *n *(%)	148 (29.7)	42 (12.7)	89 (15.5)
Current smokers, *n *(%)	223 (44.7)	121 (36.7)	237 (41.1)
Primary or lower education, *n *(%)	42 (8.5)	87 (26.2)	42 (7.1)
Secondary education, *n *(%)	129 (26.0)	144 (43.4)	244 (41.4)
College/further education, *n *(%)	141 (28.4)	67 (20.2)	186 (31.6)
Academic/polytechnic education, *n *(%)	184 (37.1)	34 (10.2)	117 (19.9)
Social class, I, n (%)	75 (15.2)	14 (4.7)	47 (8.9)
II, n (%)	134 (27.2)	37 (12.3)	99 (18.7)
IIIa, n (%)	78 (15.9)	49 (16.3)	114 (21.6)
IIIb, n (%)	35 (7.1)	43 (14.3)	109 (20.6)
IVa-c, n (%)	38 (7.7)	13 (4.3)	13 (2.5)
V-VI-VII, n (%)	132 (26.8)	144 (48.0)	147 (27.8)
**Physical activity**			
Median total score (IQR)	7440 (5400, 10316)	7082 (457511, 317)	8160 (5877, 12157)
T1 (0, 6180), *n *(%)	170 (33.9)	139 (41.1)	172 (28.9)
T2 (6180, 9690), *n *(%)	187 (37.3)	87 (25.7)	202 (33.9)
T3 (9690, 38600), *n *(%)	145 (28.9)	112 (33.1)	222 (37.2)
Mean intensity score (SD)	27.91 (10.0)	21.14 (7.2)	23.69 (8.1)
Mean duration score (SD)	709.57 (303.8)	640.43 (300.9)	779.97 (349.5)
**Blood lipids**			
Mean HDL, mmol/L (SD)	1.50 (0.4)	1.28 (0.4)	1.46 (0.4)
Low HDL, *n *(%)^a^	96 (19.1)	148 (43.8)	154 (25.8)
Median triglycerides, mmol/L (IQR)	1.08 (0.75, 1.64)	1.24 (0.88, 1.83)	0.84 (0.58, 1.20)
Raised triglycerides, *n *(%)^b^	125 (24.9)	106 (31.4)	70 (11.7)
Currently receiving cholesterol/triglyceride medication, *n *(%)	16 (3.2)	23 (6.8)	8 (1.3)
Known high cholesterol and/or triglycerides	56 (11.2)	55 (16.3)	37 (6.2)

IQR, interquartile range; HDL, high-density lipoprotein; SD, standard deviation. Social class categories adapted from Erikson *et al*. [15]. Blood lipid values are presented in mmol/L. ^a^Low HDL cholesterol < 1.04 mmol/L (< 40 mg/dL) for men and < 1.29 mmol/L (< 50 mg/dL) for women. ^b^Raised triglycerides ≥1.7 mmol/L (≥150 mg/dL) or receiving cholesterol/triglycerides lowering medication. Triglycerides can be converted to mg/dL by multiplying with a factor 88.5. HDL cholesterol levels can be converted to mg/dL by multiplying with a factor 38.6

The African-Surinamese had the highest total PA score. African-Surinamese were most often in the highest tertile of the total PA score and had higher duration scores. The European-Dutch had the highest intensity score. Hindustani-Surinamese participants had both lower intensity scores and lower duration scores than the African-Surinamese and European-Dutch participants. The Hindustani-Surinamese had lower levels of HDL cholesterol and higher levels of triglycerides than the European-Dutch. Hindustani-Surinamese participants were also most often receiving medication for high cholesterol and triglycerides. The African-Surinamese had the lowest level of triglycerides.

**Figure 1:**
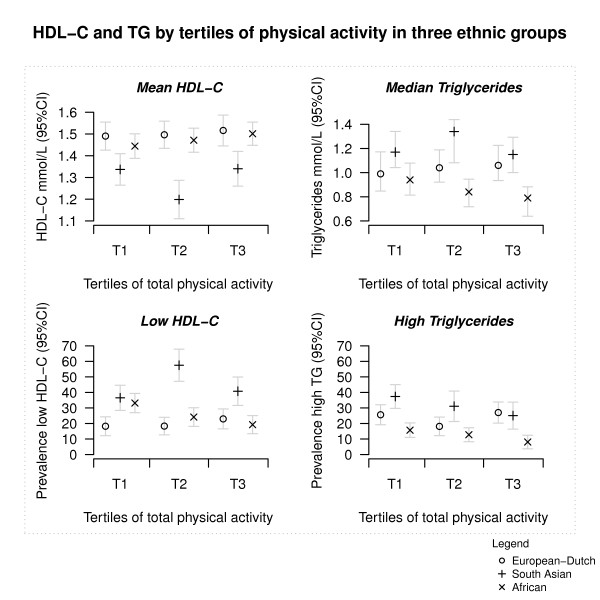
**Age-sex standardised mean HDL-C, median TG, the prevalence of low HDL-C, and the prevalence of high TG in three ethnic groups by tertiles of a total physical activity score**. Participants with known raised cholesterol or TG levels or receiving cholesterol/TG lowering medication were excluded from mean HDL-C and median TG plots. Low HDL-C was defined as < 1.04 mmol/L (< 40 mg/dL) for men and < 1.29 mmol/L (< 50 mg/dL) for women. Raised TG was defined as ≥1.7 mmol/L (≥150 mg/dL) or receiving cholesterol/TG lowering medication. TG: Triglycerides.

We first examined the patterns between tertiles of total PA with levels of HDL cholesterol and triglycerides in each of the ethnic groups (Figure 1). For the African-Surinamese group, we observed a higher mean HDL cholesterol level in the highest tertile of total PA than in the lowest tertile of total PA (beta: 0.07, 95% CI: -0.01, 0.14). We also observed a similar beneficial pattern for median triglycerides in the highest tertile of total PA compared with lowest tertile of total PA (GMR: 0.90, 95% CI: 0.80, 1.00). The prevalence of low HDL cholesterol in the highest tertile of total PA compared to the lowest tertile of total PA (PR: 0.60, 95% CI: 0.41, 0.89) and the prevalence of raised triglycerides (PR: 0.52, 95% CI: 0.28, 0.97) showed similar occurrences. The European-Dutch and Hindustani-Surinamese groups did not show such a pattern except for the prevalence of raised triglycerides in Hindustani-Surinamese, although this was not statistically significant (PR: 0.68, 95% CI: 0.43, 1.07).

The age- and sex-adjusted models for total PA showed patterns similar to those just described (Table 2). For the African-Surinamese group, the highest tertile of total PA was associated with more beneficial levels of HDL cholesterol and triglycerides, while this was not so for the European-Dutch and South Asians. These patterns in the ethnic groups persisted after further adjustment for level of education, smoking, alcohol intake or social class (data available on request). Similar results were found when total PA was substituted with MET-hours (data not shown).

**Table 2:** Association of total physical activity with high-density lipoprotein cholesterol and triglyceride levels

	European-Dutch	Hindustani-Surinamese	African-Surinamese
**Mean HDL**	**β (95% CI)**	**β (95% CI)**	**β (95% CI)**
T1	Reference	Reference	Reference
T2	0.01 (-0.08, 0.09)	-0.14 (-0.25, -0.03)	0.04 (-0.04, 0.12)
T3	0.04 (-0.05, 0.13)	0.01 (-0.09, 0.12)	0.08 (0.01, 0.16)
			
**Low HDL in percentage**	**PR (95% CI)**	**PR (95% CI)**	**PR (95% CI)**
T1	Reference = 1	Reference = 1	Reference = 1
T2	0.94 (0.57, 1.55)	1.45 (0.98, 2.14)	0.73 (0.50, 1.07)
T3	1.25 (0.76, 2.07)	1.06 (0.71, 1.58)	0.59 (0.40, 0.88)
			
**Triglycerides**^**a**^	**GMR (95% CI)**	**GMR (95% CI)**	**GMR (95% CI)**
T1	Reference = 1	Reference = 1	Reference = 1
T2	1.07 (0.95, 1.20)	1.12 (0.95, 1.33)	0.98 (0.88, 1.10)
T3	1.07 (0.94, 1.21)	1.01 (0.86, 1.19)	0.91 (0.82, 1.02)
			
**High triglycerides in percentage**	**PR (95% CI)**	**PR (95% CI)**	**PR (95% CI)**
T1	Reference = 1	Reference = 1	Reference = 1
T2	0.83 (0.54, 1.28)	0.95 (0.59, 1.54)	0.87 (0.49, 1.53)
T3	1.06 (0.69, 1.61)	0.74 (0.46, 1.18)	0.54 (0.29, 1.02)

CI, confidence interval; GMR, geometric mean ratio; HDL, high-density lipoprotein; PR, prevalence ratio(estimated with robust Poisson regression); T1-T3, tertiles of the total physical activity score. The estimates for β and the GMR are based on a restricted dataset excluding participants with known raised cholesterol and/or triglyceride levels. Estimates are adjusted for age and sex.

^a^Triglyceride levels were skewed and therefore log-transformed in the model. The values represent the GMRs compared to each reference group. Low HDL cholesterol < 1.04 mmol/L (< 40 mg/dL) for men and < 1.29 mmol/L (< 50 mg/dL) for women. Raised triglycerides ≥1.7 mmol/L (≥150 mg/dL) or receiving cholesterol/triglycerides lowering medication.

As the second step, we examined the association of the intensity score and the duration score for HDL cholesterol and triglycerides in each ethnic group. Intensity of activity was associated with HDL cholesterol and triglycerides (Table 3). One standard deviation increase in intensity score was associated with a higher HDL cholesterol level in the European-Dutch group (beta: 0.06, 95% CI 0.03, 0.10), which we also observed in the African-Surinamese group (beta: 0.06, 95% CI: 0.02, 0.10), but not in the Hindustani-Surinamese group (beta: -0.03, 95% CI: -0.09, 0.04). We observed lower levels for triglycerides, which were associated with one standard deviation increase in intensity in all ethnic groups.

**Table 3:** Association of physical activity intensity and duration scores with high-density lipoprotein cholesterol and triglyceride levels

Physical activity Intensity score	European-Dutch	Hindustani-Surinamese	African-Surinamese
HDL-C, β (95% CI)	0.06 (0.03, 0.10)	-0.03 (-0.09, 0.04)	0.06 (0.02, 0.10)
TG, GMR (95%CI)	0.93 (0.88 0.97)	0.94 (0.86 1.04)	0.96 (0.91 1.01)
			
**Physical activity Duration score**			
HDL-C, β (95% CI)	0.00 (-0.05, 0.06)	-0.01 (-0.09, 0.07)	-0.05 (-0.09, -0.00)
TG, GMR (95%CI)	0.96 (0.89 1.04)	1.04 (0.92 1.18)	1.01 (0.95 1.08)

The estimates are based on a restricted dataset excluding participants with known raised cholesterol and/or triglyceride levels or on lipid lowering medication. The models are adjusted for age, sex and mutual adjustment for the intensity, duration, and frequency scores. Triglyceride levels were skewed and therefore log transformed in the model; the estimates are back transformed to obtain the GMR. GMR: Geometric Mean Ratio. TG: Triglycerides

The duration score was not associated with HDL cholesterol levels for the European-Dutch and Hindustani-Surinamese. The pattern of the duration score and HDL cholesterol level appeared to differ in the African-Surinamese group, although a likelihood test for ethnicity interaction did not confirm this. There was also a lack of association of the duration score with triglycerides in all ethnic groups.

## Discussion

### Main findings

The association of *total *PA with HDL cholesterol and triglyceride levels only occurred in the African-Surinamese group. The intensity component of PA was consistently associated with the triglyceride levels in all ethnic groups and with HDL cholesterol levels in the European-Dutch and African-Surinamese groups. The duration of PA was not associated with any outcome measure except the HDL cholesterol level in the African-Surinamese group.

### Limitations

Many multi-ethnic studies encounter language difficulties. The Surinamese groups of South Asian and African descent living in the Netherlands that were included in this study originate from the former Dutch colony of Suriname, where the official language is Dutch. These groups therefore understand the Dutch language well, which can be considered a strength of this study. However, as in many observational studies, our PA measures were based on self-reported data, which might be influenced by a cross-cultural difference in reporting of PA.

The additional influence of diet on blood lipids may play a role in our multi-ethnic study population. We were unable to look further into this relationship because the necessary data for this study population was unavailable. Future studies should take this into account.

### Discussion of main findings

In our study, total PA was associated with HDL cholesterol and triglyceride levels in the African-Surinamese group, but not in the European-Dutch and Hindustani-Surinamese groups. This lack of association with total PA in the European-Dutch group was particularly surprising. Total PA (from multiple domains) has been associated with more beneficial levels of HDL cholesterol and triglycerides in other study populations of European descent [17,18]. Nevertheless, there are other patterns, for example, a lack of association of PA with HDL cholesterol levels in the European population in Newcastle upon Tyne [7].

The explanations for these different findings are unclear. However, it is very likely that differences in study methods may contribute to differences in the findings. For example, Fransson *et al*.'s study used a single-scale PA self-report measurement (with either 15 categories or 3 categories) for each domain that did not identify the components of duration, frequency, and intensity separately [17]. Hayes *et al*. measured sports and exercises by duration in moderate or vigorous intensity (based on METs), while categorising walking and cycling by distance only [19]. Therefore, intense walking and cycling might have been missed, which could possibly diminish the association in the study sample. In our study, however, the participants could report every component (duration, frequency, and intensity) for every activity, and we also found a lack of association of our total PA score with HDL levels. Alternatively, it might be that the absolute level of PA was too low to adequately model the variation in blood lipids (resulting in wider confidence limits) in this study sample, particularly in the European-Dutch and Hindustani-Surinamese ethnic groups.

Therefore, we also focussed on the contributions of the components of PA. Because we took the intensity and duration of PA into account, associations that were more similar across the ethnic groups became visible. This is in line with other multi-ethnic studies that found beneficial associations of the HDL cholesterol level with intensity of activity, although most evidence comes from studies including only sports and exercise in leisure time PA and not total PA [6]. Triglyceride levels were not associated with intensity of activity in African Americans in the study by Monda *et al*. [20]. The explanation given was the more favourable lipid profile of the African participants. We also observed a more favourable triglyceride level among the African-Surinamese in our study; however, there was also an association between intensity of activity and triglyceride level in this population. Other lifestyle factors, for instance the influence of diet on triglyceride level, could be responsible for this different association of physical activity between these groups.

The only instance where we were unable to see an association with intensity of activity was in the HDL cholesterol level of the Hindustani-Surinamese group. We believe this was more likely due to the low levels of reported PA in this group, resulting in less precise estimates and wider confidence intervals. Low levels of PA in populations of South Asian descent have been reported before [21]. Even though we did not observe an association, there is no reason to doubt the beneficial effect of intense PA on the blood lipids of South Asians. For example, Misra et al. found a relationship between heavy activity and HDL cholesterol in Asian Indian men [22]. In another example, a 6-week training programme for female Indian boxers showed a favourable effect on their HDL cholesterol levels [23].

The intensity of the activity contributed more to the association in the HDL cholesterol and triglyceride levels in our multi-ethnic study population than the duration of activity. We observed this lack of beneficial association of duration of activity with blood lipids for the whole study population (there was an indication of an opposite association in the African-Surinamese group, although it was not statistically significant in an overall test for interaction). One explanation for this lack of association is that most of the duration of activity in our study population comes from less intense activities, which have a weaker relationship with blood lipids. For example, Westerterp and Plasqui's work show that vigorous PA is often of short duration and, therefore, most of the duration of total activity comes in the form of less intense activities [24]. The observation that duration of activity seems inversely related to HDL in African-Surinamese, might be related to this explanation. While intensity contributed more to the association with blood lipids in our study population than duration, there is evidence in randomized controlled trials that, besides an effect of intensity on blood lipids, the amount of activity (distance) and duration is positively related to the blood lipid profile [25,26]. While the amount is closely related to duration, the duration and intensity of activity in these trials were generally greater than in our study population (for example, brisk walking or jogging a distance of 10 or 15 km three times a week).

The results of this study suggest that the inconsistent association with the total PA measure might be due to the varying composition of the PA between ethnic groups, and thus might relate to the measurement of PA. These findings are very relevant to other multi-ethnic populations living in a different context, especially when patterns of PA (in terms of duration and intensity) are markedly different between population groups. In addition to a measure of total PA or energy expenditure, separate presentations of the composition of the PA, especially intensity of activity, is needed in PA research.

## Competing interests

The authors declare that they have no competing interests.

## Authors' contributions

All authors have made substantial contributions to conception and design, interpretation of data, revising it critically for important intellectual content, and have given final approval of the version to be published.
